# Does normobaric hyperoxia increase oxidative stress in acute ischemic stroke? A critical review of the literature

**DOI:** 10.1186/s13618-015-0032-4

**Published:** 2015-08-25

**Authors:** John Weaver, Ke Jian Liu

**Affiliations:** Department of Pharmaceutical Sciences, College of Pharmacy, BRaIN Imaging Center, MSC10 5620, 1 University of New Mexico Health Sciences Center, Albuquerque, NM 87131 USA; Center of Biomedical Research Excellence, College of Pharmacy, University of New Mexico Health Sciences Center, Albuquerque, NM 87131 USA; Department of Neurology, University of New Mexico Health Sciences Center, Albuquerque, NM 87131 USA

## Abstract

Stroke, one of the most debilitating cerebrovascular and nuerological diseases, is a serious life-threatening condition and a leading cause of long-term adult disability and brain damage, either directly or by secondary complications. Most effective treatments for stroke are time dependent such as the only FDA-approved therapy, reperfusion with tissue-type plasminogen activator; thus, improving tissue oxygenation with normobaric hyperoxia (NBO) has been considered a logical and potential important therapy. NBO is considered a good approach because of its potential clinical advantages, and many studies suggest that NBO is neuroprotective, reducing ischemic brain injury and infarct volume in addition to improving pathologic and neurobehavorial outcomes. However, increased reactive oxygen species (ROS) generation may occur when tissue oxygen level is too high or too low. Therefore, a major concern with NBO therapy in acute ischemic stroke is the potential increase of ROS, which could exacerbate brain injury. The purpose of this review is to critically review the current literature reports on the effect of NBO treatment on ROS and oxidative stress with respect to acute ischemic stroke. Considering the available data from relevant animal models, NBO does not increase ROS or oxidative stress if applied for a short duration; therefore, the potential that NBO is a viable neuroprotective strategy for acute ischemic stroke is compelling. The benefits of NBO may significantly outweigh the risks of potential increase in ROS generation for the treatment of acute ischemic stroke.

## Introduction

Stroke is one of the most common causes of death and long-term disability of adults, and as the brain is highly sensitive to hypoxia insufficient brain oxygen plays a crucial role in the primary and secondary events leading to neuronal cell death and damage [1–3]. Currently, systemic thrombolysis with tissue-type plasminogen activator remains the only FDA-approved reperfusion strategy for acute ischemic stroke [4], which must be given during the early onset of stroke and delayed therapy significantly increases the risks of intracranial hemorrhage [5]. For this reason, improving tissue oxygenation with oxygen therapy has been considered a logical and potentially important therapy for acute ischemic stroke. As adequate cerebral oxygen supply is crucial to neuron survival, the rational for this approach is to restore cellular oxygenation of damaged neurons, particularly in the penumbra, compromised after ischemic stroke, and stimulate those damaged neurons to function normally. Specifically, normobaric hyperoxia (NBO) may be especially useful for improving brain tissue oxygen [6–9]. NBO is considered a good approach because of its potential clinical advantages. It is inexpensive, non-invasive, can be widely available and simple to administer to acute stroke patients by medical staff in a broad range of conditions, including by paramedics or at home. In accord, there are many studies that suggest that NBO is neuroprotective and can reduce acute ischemic brain injury, reduce infarct volume and improve pathologic and neurobehavorial outcomes [10–26].

Electron paramagnetic resonance (EPR) oximetry studies highlight the importance of monitoring tissue oxygen during acute ischemic stroke and NBO treatment [13, 17, 27]. EPR studies have shown that NBO treatment applied during ischemia restores penumbra tissue back to or above preischemic levels [13, 17, 27], while NBO during reperfusion increases penumbra tissue oxygen to twice preischemic levels [17]. As the ischemic penumbra is considered the main target of oxygen therapy, increased oxygen status in the penumbra can improve metabolic status and survival of tissue, but more importantly may also trigger the generation of reactive oxygen species (ROS) and oxidative damage when brain tissue oxygen levels are too high or too low [28, 29]. Thus, a major concern with NBO in acute ischemic stroke is the potential increase in the generation of ROS, thereby exacerbating brain injury [7]. In this review, we will discuss the history of oxidative stress with respect to ischemia and hyperoxia, and the existing controversy surrounding NBO and oxidative stress, particularly from relevant experimental models. We will also summarize the current literature on the impact of NBO treatment on oxidative stress in acute focal ischemia stroke animal models, and if potential NBO induced oxidative stress offset the suggested neuroprotective properties of NBO.

### Oxidative stress in ischemia/reperfusion injury and the evolution of oxygen treatment

The concept of oxidative damage results from the production of ROS and byproducts at rates which exceed the ability of natural antioxidant defense mechanisms to detoxify these deleterious products, likely promoting damage. It has been suggested that ROS and oxidative stress is an important mechanism in ischemic/reperfusion injury when ischemia is long and when conditions allow for oxygen to be restored to the ischemic tissue [30–34]. Reintroduction of oxygen to ischemic tissue may trigger a series of harmful reactions leading to the production of ROS and as a consequence inflammation and oxidative damage rather than the restoration to normal function. Accordingly, oxygen therapy or hyperoxia may contribute to oxidative ischemic/reperfusion injury by introducing a compliment of ROS and oxidative stress.

The idea that hyperoxia causes oxidative stress dates back to a novel study hypothesizing that the similarities between the toxic effects of oxygen toxicity and gamma radiation in mice were associated with the production of ROS [35]. Many brain specific studies, beginning in the early 1960s, would then provide supporting evidence of the involvement of ROS in oxygen toxicity by hyperoxia either by an increase in ROS, increase in lipid peroxidation, or circumstantial evidence from studies using agents to effect antioxidant mechanisms and pretreatments to make animals tolerant to hyperoxia [36–53]. However, in a number of these studies, hyperbaric oxygen (HBO) treatment, 100 % oxygen at a pressure greater than 1 atm absolute, was used as the model for hyperoxia and oxygen toxicity. HBO will not be discussed in detail herein, but in clinical trials for ischemia, HBO has shown mixed outcomes [54–58]. Subsequently, recent review articles have provided detailed findings on the effects of HBO on ischemia and oxidative stress in animal studies [7, 59–61]. While controversial, these reviews have emphasized that HBO is associated with increased oxidative stress in several studies, mainly linked to excessive duration of exposure along with high concentrations and pressures. Likewise, differences in experimental conditions and trial design in clinical trials have been recognized as a contributing factor for the mixed results and more controlled studies are needed to determine the effectiveness of HBO treatment.

The risks of NBO induced oxidative stress are also suggested to be associated with duration or prolonged exposure [21, 62, 63]. In addition, the degree of hyperoxia, composition of the inhaled gas mixture, age (especially neonatal vs adult), health and species of animal may play a significant role in determining the therapeutic potential of NBO [37, 48, 64–67]. Thus far, there are a small number of clinical trials using NBO as a potential treatment for ischemic stroke, and mostly all have shown either a potential benefit or no effect [15, 16, 20, 22, 24] with one trial cautioning the use of NBO when given for 24 h [68]. To date, no trials have provided evidence that NBO was detrimental. One clinical study in particular has shown NBO to be neuroprotective when applied within 12 h after the onset of stroke, and these positive results, despite the small sample size, call for further studies to investigate the optimal time window for NBO duration and effects in different types of stroke [15]. Even though neurological outcomes are promising in clinical trials of NBO, the uncertainty of potential damage from NBO induced oxidative stress which may aggravate ischemia/reperfusion injury remains a concern. However, many animal studies have presented findings on the use of NBO treatment in focal acute ischemia/reperfusion injury models, and its effects on the formation of ROS, reactive nitrogen species (RNS) or increased oxidative stress, if any, were reported. These studies are summarized in the following sections (Table 1).

**Table 1:** Summary of neurological outcomes in acute focal brain ischemia NBO studies that measure oxidative stress

Model	NBO intervention	Oxidative stress results	Neurological outcome results	Ref.
MCAO (2 h) and reperfusion (60 min ) in rats	NBO (100 % O_2_) during MCAO and reperfusion	NBO did not increase HO-1 induction and protein carbonyl formation (no significant differences between groups)	NBO significantly reduced total infarct volume (70 %) and cortex infarct volume (92 %), but not significantly in the striatum. No significant differences in BBB damage.	[12]
MCAO (1,2,3, or 4 h) in rats	NBO (100 % O_2_) during MCAO	NBO did not increase markers for O_2_·^−^ generation (Het) (no significant differences between groups)	NBO groups improved neurological scores and had significantly smaller infarct volumes after 1,2, and 3 h MCAO	[14]
MCAO (90 min) and reperfusion (90 min) in rats	dNBO (95 % O_2_) during MCAO or rNBO during reperfusion	dNBO (not rNBO) significantly reduced 8-OHdG production. dNBO and rNBO did not affect O_2_·^−^ generation (Het).	NBO during MCAO significantly reduced infarct volume (40 %). NBO during reperfusion reduced infarction volume (15 %: not significant)	[17]
MCAO (90 min) and reperfusion (22.5 h) in WT and gp91phox-KO mice	NBO (95 % O_2_) during MCAO	NBO significantly inhibited gp91phox expression, NADPH oxidase activity, and MMP-9 induction in WT	NBO treatment and gp91phox-KO significantly reduced BBB damage. Inhibition of gp91phox may be an important mechanism underlying NBO-afforded BBB protection	[23]
MCAO (90 min) and reperfusion (90 min) in mice	dNBO (100 % O_2_) during MCAO or rNBO (100 %) during reperfusion	dNBO significantly decreased levels of 4HNE, NT, 8-OHdG oxidation. No significant differences with rNBO	dNBO significantly reduced infarct volume (~50 %) and improved sensorimotor scores. rNBO worsened the ischemic lesion, but no significant difference in sensorimotor scores	[27]
BCAO (15 min) and blood pressure (50 mmHg) reduction in rats	NBO (100 % O_2;_ 3 h) after BCAO	NBO did not lead to enhanced H_2_O_2_ or ROS production (no significant differences between groups)	No significant differences in caudoputaminal and neocortical damaged neurons score or percent of hippocampal necrotic neurons	[32]
MCAO (90 min) and reperfusion (22.5 h) in rats	NBO (95 % O_2_) given during MCAO	NBO significantly inhibited gp91phox expression and NADPH oxidase activity	NBO significantly reduced MRI ADC lesion volume (37 %)	[67]
MCAO (90 min) and reperfusion (22.5 h) in rats	iNBO (100 % O_2_), sNBO, nNBO, or cNBO^a^	iNBO and nNBO groups significantly decreased O_2_·^−^ production (Het)	iNBO significantly reduced infarct volume (34 %) equivalent to nNBO. sNBO did not decrease infarct volume and cNBO did not improve protection over iNBO or nNBO	[69]
MCAO (90 min) and reperfusion (22.5 h) in rats	NBO (100 % O_2_) given for 2,4 or 8 h 30 min after MCAO	NBO (2,4 and 8 h) significantly reduced 8-OHdG and gp91phox production (greatest reduction with 8 h NBO)	NBO (2,4 and 8 h) significantly reduced total infarct volume and infarct volume in the cortex and subcortex (8 h NBO offered the greatest efficacy)	[70]
MCAO (90 min) and reperfusion (22.5 h) in rats	NBO (95 % O_2_) given during MCAO	NBO significantly inhibited gp91phox expression, NADPH oxidase activity, and MMP-9 induction	NBO significantly decreased BBB permeability coefficient (MRI) and brain edema	[71]
MCAO (90 min) in WT and SOD2-KO mice	NBO (100 % O_2_) (60 min) started 25 min after MCAO	NBO significantly decreased O_2_·^−^ generation (Het) in WT mice.	NBO does not affect infarct size in SOD2-KO mice and the effect of NBO on infarct size in WT mice was not presented	[72]
MCAO (10,30,60 or 90 min) in rats	NBO (95 % O_2_) given during MCAO	NBO significantly delayed and attenuated ·NO production (NOx^−^:nitrite plus nitrate) and significantly reduced 3-NT formation	NBO significantly decreased total infarct volume, particularly in the cortex, but not significantly in the striatum	[73]

*MCAO* middle cerebral artery occlusion, *HO-1* heme oxygenase-1, *O*_*2*_ *·* ^*−*^ superoxide, *Het* dihydroethidium, *dNBO* during MCAO, *rNBO* during reperfusion, *4HNE 4-*hydroxynonenal, *NT* nitrotyrosine, *8-OHdG* 8-hydroxy-2′–deoxyguanosine, *BCAO* bilateral carotid artery occlusion, *WT* wild-type, *KO* knock-out, *iNBO* Intermittent, *sNBO* Short*, nNBO* normal or continuous, *cNBO* combination, *·NO* nitric oxide

^a^ see reference for details

### The Effect of NBO on oxidative stress in focal acute ischemia animal models

Initially, we will review a series of studies in which NBO is given shortly after the onset of focal acute ischemia and the impact NBO has on oxidative stress under these conditions. In 1991, a study by Agardh CD et al. found that NBO following brief 15 min periods of ischemia, by bilateral carotid artery occlusion, did not lead to enhanced H_2_O_2_ production and that there was no indication that postischemic oxygen supply altered the productions of ROS [32]. Singhal AB et al. indicated that NBO treatment during a filament model of transient middle cerebral artery occlusion (MCAO) ischemia/reperfusion did not increase oxidative stress as measured by heme oxygenase-1 induction and protein carbonyl formation [12]. The same group also indicated that NBO did not increase markers for superoxide (O_2_·^−^) generation, measured by dihydroethidium (Het) fluorescence [14]. In these studies, no significant difference was observed between NBO and non-treatment animals suggesting that NBO neither increased nor decreased oxidative stress or ROS formation.

In a series of studies by the Liu group, it was shown that NBO treatment, given intermittently or continuously, during MCAO (90 min) either had no effect or reduced O_2_·^−^ generation as measured by the Het fluorescence [17, 69]. The generation of 8-hydroxy-2′–deoxyguanosine (8-OHdG), a biomarker for oxidative DNA damage, was also reduced compared to non-treatment animals [17, 70]. Alongside, an increase in ROS and 8-OHdG was not observed in the contralateral hemispshere of the ischemic animals given NBO suggesting that the brain is more tolerable to hyperoxia [17]. Follow up studies indicated that NBO inhibited the upregulation of nicotinamide adenine dinucleotide phosphate (NADPH) oxidase catalytic subunit, gp91^phox^, along with the expression of mRNA and protein of gp91^phox^. It was concluded that NBO reduced NADPH activity, a principal enzyme responsible for O_2_·^−^ generation [67, 69, 71], and a subsequent study would then suggest that gp91^phox^ containing NADPH oxidase was an important mechanism in NBO-afforded neuroprotection in ischemia using gp91^phox^ knock-out mice [23]. Of note, Yaun Z et al. also demonstrated that NBO for 8 h starting during ischemia and continued during reperfusion was the most effective at decreasing oxidative stress compared to shorter durations [70]. Likewise, a study by the Veltkamp group indicated that O_2_·^−^ formation, again measured by Het, significantly decreased in mice treated with NBO during ischemia compared to room air controls, while superoxide dismutase (SOD) 2 knock-out mice, genetically susceptible to ROS-induced brain injury, were unaffected by NBO suggesting that NBO does not appear to promote damage associated with ROS [72].

Similar reports also support the hypothesis that NBO does not increase ROS production or oxidative stress when given during reperfusion. In the Liu S et al. study reviewed above, NBO given during reperfusion (90 min) instead of ischemia exhibited no difference in ROS and 8-OHdG production compared to control animals [17]. Rink C et al. showed that NBO given during reperfusion (90 min) did not significantly increase histochemical markers of oxidative stress, 4-hydroxynonenal (4HNE) (a measure of lipid peroxidation), the protein nitrotyrosine (NT) and 8-OHdG oxidation, in the ischemic hemisphere compared to room air controls [27]. Of note, in the same study, Rink C et al. showed that NBO given during MCAO decreased levels of 4HNE, NT and 8-OHdG oxidation in the ischemic hemisphere compared to room air controls [27] similar to previous studies [17, 70].

The effect of NBO on RNS has also been investigated in an acute ischemia small animal model. In an earlier study by Yuan et al., they showed that NBO treatment during MCAO delayed and attenuated nitric oxide (·NO) production via the measurement of nitrite plus nitrate (NOx^−^), presumably by inhibiting neuronal nitric oxide synthase (nNOS) [73]. Specifically, ischemia caused a rapid production of ·NO 10 min after stroke onset and NBO delayed this up to 30 min and decreased the total amount of NOx^−^ produced. NBO also inhibited the oxidative stress marker, 3-NT, similar to the nNOS inhibitor, 7-nitroxindazole, compared to untreated rats [73].

Collectively, all of these studies suggest that short duration of NBO treatment does not promote additional oxidative stress in acute ischemic animal models and may potentially decrease oxidative stress or ROS/RNS depending on experimental conditions (Table 1). As a majority of acute ischemia experimental studies mostly focus on acute 24 or 48 h outcomes, the question can be raised that more time may be needed for secondary events to develop with NBO treatment and thus we may be monitoring different aspects of oxidative stress mechanisms [38]. For clinical translation, it may be advantageous to show that NBO does not promote oxidative stress outside the acute 48 h window or at least that any effect is extraneous, which current studies, herein, support and the long term benefits of NBO treatment suggests [26]. That said, the mechanisms underlying the effect of NBO on oxidative stress are still not fully understood and further investigations are needed.

### NBO afforded neuroprotection in focal acute ischemia animal models

Seeing that a large pool of evidence suggests that NBO does not increase oxidative stress in acute ischemic stroke or may possibly decrease oxidative stress dependent on experimental condition, many of these studies also insinuate that NBO affords neuroprotection or does not worsen neurological outcomes in acute ischemic stroke. Uninterrupted or continuous NBO given shortly after ischemia decreased infarct volume significantly in many of the studies [12, 17, 27, 70, 72, 73]. It was even demonstrated in a novel study by Liu et al. that intermittent NBO and a combination of intermittent and continuous NBO, given during MCAO, significantly reduced infarct volume [68]. Similarly, measurements of lesion volume, a compliment to infarct volume, by MRI apparent diffusion coefficients was also significantly reduced in a study wherein NBO was given during MCAO [67]. This study was in agreement with several clinical studies which demonstrate improvement of clinical deficits and MRI abnormalities [15, 16, 20, 24]. Additionally, NBO given during MCAO was shown to reduce BBB damage and brain edema, a measure of oxidative damage [70], and more recently, Sun et al. indicated that NBO therapy had no effect on infarct volume in SOD2 knockout mice, again suggesting that NBO did not appear to promote damage inflicted by ROS [72].

The neuroprotective benefits of NBO have also been investigated when given during reperfusion. In the study conducted by Liu S et al., in which NBO given during ischemia reduced infarct volumes, it was determined that NBO given during reperfusion had no significant effect on infarct volume [17]. However, in a similar study, Rink et al. indicated that while NBO given during ischemia reduced infarct volume, NBO given during reperfusion significantly exacerbated the ischemic lesion. [27]. In the same study NBO given during reperfusion had no significant effect on sensorimotor assessment and did not exhibit elevated levels of oxidative stress suggesting other factors may be involved. The authors commented that timing of NBO treatment is crucial in determining the benefits of NBO, and the mixed outcomes of these studies warrants further investigation. Nonetheless, in a study investigating the relationship between outcome and NBO duration, Yuan Z et al. showed that NBO given for 8 h, started during MCAO and continued during reperfusion, offered greater efficacy at reducing infarct volume compared to NBO given for 2 and 4 h [71]. In this study, NBO treatment started during ischemia and extended into the reperfusion window seemingly improved the benefits of NBO.

In addition to infarct volume, neurological score assessments have been used to address NBO afforded neuroprotection or damage in studies investigating NBO treatment and acute ischemic stroke. In a study wherein NBO was given during ischemia, Kim et al. showed that NBO did improve neurological scores [14], while Agardh CD et al. indicated that NBO given following brief ischemia (15 min) did not enhance or decrease damage as measured by neurological damage scores [32]. Conversely, two studies have demonstrated that NBO preconditioning, although unbefitting for ischemic stroke patients, could prevent ischemic/reperfusion injury or increase ischemic tolerance [21, 25]. Both studies indicated that NBO preconditioning decreased infarct volume, significantly, while Chen et al. [25] showed that NBO also significantly improved neurological scores compared to controls. It was suggested that overactivation of antioxidant activities [21] and the hypoxia-inducible factor signaling pathway [25] may play a role in ischemic tolerance or protection. Given that NBO preconditioning was neuroprotective, it was suggested that preconditioning may be beneficial for those at risk of stroke or in surgeries involving selective cerebral perfusion [21, 25, 74, 75], but the potential neuroprotective effects of NBO preconditioning need to be investigated further.

Worthy of note, a study conducted by Shin HK et al. indicated that NBO treatment, in a distal MCAO model, reduced infarct volume and improved neurological function in normal wild-type mice compared to mice with endothelial dysfunction [76]. It was suggested that the neuroprotective effects of NBO are critically dependent on normal endothelial function possibly linked to RNS production from endothelial NOS, but production of RNS was not investigated. Notwithstanding, a majority of the studies in this section support the hypothesis that NBO treatment is neuroprotective or does not stimulate additional damage with respect to acute ischemia/reperfusion injury. One study, by Esposito et al., even supports the neuroprotective effects of NBO long term (up to 2 weeks) in an animal model [26], but if these effects last for months or years in human patients warrants further examination. It is also suggested that NBO may widen the time window for reperfusion [14] and for combination treatment with other neuroprotective therapies [9], but clearly many investigators agree that because of considerable differences in clinical studies, firm conclusions are difficult to make and more studies are needed to confirm neuroprotection in humans as those observed in animal studies, herein. The protective mechanisms of NBO remain misunderstood and controversial [77], but it seems well documented that NBO, if applied shortly after acute ischemia, may provide neuroprotection particularly when NBO is shown to reduce or not influence oxidative stress.

### The effect of NBO on oxidative stress in other experimental models

Although the above studies suggest that NBO may be neuroprotective in acute ischemic models with no adverse effects related to oxidative stress, the present controversy surrounding the association between NBO and oxidative stress may result from conflicting results in various *in vitro*, *ex vivo* and *in vivo* experimental models and diseases states. We will first review the relationship between NBO and oxidative stress in *in vitro and ex vivo* models. Cell culture reoxygenation studies which mimic hyperoxia conditions similar to NBO show an increase in intracellular ROS (O_2_·^−^, hydrogen peroxide (H_2_O_2_), hydroxyl radicals, and potent oxidants such as peroxynitrite) and increased oxidative stress compared to controls [78–80]. *Ex vivo* studies of tissue from the lungs, brain, liver, and kidney of NBO exposed animals also indicate an increase in oxidative stress with each organ responding differently to NBO and severity of injury being time, dose and exposure dependent [62–64, 80–82]. Taken together, these *in vitro* and *ex vivo* studies strongly associate NBO with increased oxidative stress, but care should be taken when interpreting these results as the duration of hyperoxia or NBO exposure is significant compared to suggested treatment conditions for acute ischemia *in vivo* [11]. Furthermore, hyperoxia conditions in *in vitro* studies exposes the cells to much higher oxygen concentration (~100 % directly, or 760 mmHg) than *in vivo* conditions where actual tissue oxygen level is only about 30–40 mmHg [13, 17, 27].

The effect of NBO on oxidative stress in permanent ischemic models has also added to the controversy surrounding NBO treatment. A study using Mongolian gerbils indicated that NBO (as short as 60 min) given 15 min after bilateral carotid occlusion resulted in significant production of lipid peroxidation measured by pentane production and that animals given NBO, for 3–6 h, displayed significantly higher mortality compared to control animals [83]. It was suggested that the brain was the likely site of pentane production, but not confirmed. In a more recent study, Geng et al. indicated that NBO (given for 6 h) after permanent MCAO had no significant effect on infarct volume, neurological deficit or ROS production measured via H_2_O_2_ formation. [84]. In addition, infarct volume was significantly reduced when NBO was given 45 min after MCA coagulation, while NBO (given for 60 or 120 min) had no significant effect on infarct volume in a permanent MCAO model [85]. The same study indicated that the benefits of NBO were dependent on the extent and location of permanent ischemia, which may explain the differences in results between studies [85]. Consequently, more controlled studies are needed to evaluate the effects of NBO in permanent ischemia.

Moreover, several studies have investigated the effect of NBO and oxidative stress in brain diseases such as traumatic brain injury (TBI) and multiple sclerosis. Thus far, no studies have shown preliminary evidence of increased oxidative stress from NBO in TBI models; alongside, there is supporting evidence of possible benefits in animal models [86, 87]. It is emphasized that results are somewhat controversial, but clinical TBI studies have shown no impairment from NBO as well [86, 87]. Similarly, a recent study indicated that NBO did not exacerbate oxidative stress injury or neurological outcomes in a collagenase-induced intracerebral hemorrhage model [66]. Given that NBO did not worsen outcomes related to TBI, the study suggests that NBO, if proven to be beneficial in ischemic stroke, could be started before a definitive diagnosis is made to distinguish between ischemic versus hermorrhagic stroke [88]. Finally, a novel study on multiple sclerosis showed that NBO, whether brief (1 h) or continued for 7 days did not reveal evidence of increased O_2_·^−^ production or oxidative damage when compared to animals breathing room air, and that NBO therapy may be surprisingly beneficial as a treatment for multiple sclerosis that had not been noticed previously [89].

The results of the studies presented in this section highlight the mixed results and controversy surrounding NBO, oxidative stress and its effectiveness as treatment in several disease models. Differing experimental conditions, mainly duration of exposure is attributed to the observed increases in oxidative stress and varying results, further supporting the hypothesis that long duration of NBO would likely produce ROS. However, these studies are dissimilar to acute ischemia models which theorized that early timing and short durations of NBO may minimize deleterious effects as shown in many acute ischemia animal studies, herein; thus, making NBO a potential neuroprotective treatment for several brain diseases including acute ischemic stroke.

## Conclusions

As a potential treatment for acute ischemic stroke, NBO has distinct advantages over pharmaceutical drugs such as the ease of diffusibility across the blood–brain barrier and the mechanism of action may be via multiple pathways. It is suggested that high concentrations of oxygen are well tolerated; however, decades of research have cautioned the possible harmful effects of oxygen treatment and oxidative damage. The claim is reasonable considering the oxidant radical damage hypothesis which states that increased oxygen availability produces increased ROS and oxidative stress. Nonetheless, considering the available data from relevant animal models that NBO does not increase ROS or oxidative stress if applied for a short duration, the potential that NBO is a viable neuroprotective strategy for acute ischemic stroke is compelling. The benefits of NBO may outweigh the risks of potentially enhanced ROS generation even if deemed clinically relevant. If we can manage the variables controlling oxygen delivery for the best possible clinical outcome, patients could benefit from receiving NBO at the earliest possible time. The risk to benefit ratio of oxygen therapy must be balanced to determine the safest plan for treatment of acute ischemic stroke.

## Abbreviations

NBO: Normobaric hyperoxia
EPR: Electron paramagnetic resonance
ROS: Reactive oxygen species
HBO: Hyperbaric oxygen
RNS: Reactive nitrogen species
MCAO: Middle cerebral artery occlusion
O_2_·^-^: Superoxide
Het: Dihydroethidium
8-OHdG: 8-hydroxy-2′–deoxyguanosine
NADPH: Nicotinamide adenine dinucleotide phosphate
SOD: Superoxide dismutase
4HNE: 4-hydroxynonenal
NT: Nitrotyrosine
·NO: Nitric oxide
NOx^−^: Nitrite plus nitrate
NOS: Nitric oxide synthase
H_2_O_2_: Hydrogen peroxide
TBI: Traumatic brain injury
